# An Exploratory Clinical Study of Structured Psychological Intervention for Menopausal Symptom Management

**DOI:** 10.7759/cureus.107380

**Published:** 2026-04-20

**Authors:** Emma Barwick, Julia Cox, Tracey LC Wilson, Victoria Turner, Anwesha Borah

**Affiliations:** 1 Department of Holistic Healing, Belief Coding Cognitive Rewiring, Stockton-on-Tees, GBR; 2 Department of Pharmacy, Belief Coding Cognitive Rewiring, Stockton-on-Tees, GBR; 3 Department of Neuroscience and Biotechnology, Satani Research Centre, Ahmedabad, IND

**Keywords:** cognitive symptoms, menopause, psychological well-being, structured psychological intervention, symptom severity

## Abstract

Background: Menopause is a multifaceted life transition marked by interacting physiological, psychological, and sociocultural changes. Symptom burden varies widely and is not fully explained by hormonal decline alone. Persistent psychological and cognitive symptoms highlight the need for integrative approaches that address underlying beliefs and emotional processes.

Objective: This study evaluated the effects of a structured, non-pharmacological psychological intervention targeting emotion regulation and cognitive appraisal on psychological, cognitive, and physical symptoms associated with perimenopause and menopause and assessed immediate and short-term outcomes.

Methodology: A mixed-methods, exploratory, repeated-measures design was employed. Sixty-four women aged 39-60 years with moderate-to-severe menopausal symptoms participated in a single standardised therapeutic session. Symptom severity across 19 domains was assessed at baseline, immediately post-intervention, and at one- and four-week follow-up using a study-specific 19-item numerical rating scale (0-10). Quantitative analyses included paired t-tests and effect size estimation, complemented by qualitative self-report data.

Results: Significant reductions were observed across all symptom domains following the intervention. Psychological and cognitive symptoms demonstrated large to very large effect sizes, with improvements sustained over follow-up. Physical and vasomotor symptoms showed moderate effect size magnitudes. 89% of assessed symptoms demonstrated large or very large effect sizes (Cohen’s d ≥ 0.5).

Conclusion: The findings suggest that a structured, non-pharmacological psychological intervention may offer meaningful short-term benefits as an adjunctive approach for managing menopausal symptoms. Further randomised controlled studies are warranted to establish causal efficacy and long-term outcomes.

## Introduction

Menopause is an inherent change in the body, which most often occurs between the ages of 45 and 55 years, and it is a medical occurrence, the fundamental feature of which is the absence of menstruation during 12 continuous months [1]. In addition to this definition, menopause is a complicated life transition that is associated with interrelated physiological, psychological, and social alterations that are not limited to reproductive ageing [2]. Menopausal symptoms are heterogeneous in terms of their prevalence, type, and severity across individuals [3]. Typical physical symptoms of menopause are vasomotor, like hot flushes and night sweats, musculoskeletal, fatigue, and sleep abnormalities [4]. In addition to these physical changes, most women have psychological and cognitive symptoms, such as anxiety, depressive symptoms, irritability, mood swings, decreased libido, and subjective cognitive impairment commonly known as brain fog [5]. These are long-term symptoms that can last over a number of years and are linked to a loss of daily functioning and occupational performance, as well as a decrease in quality of life [6].

Even though a reduction in oestrogen and progesterone is the primary part of the menopausal transition, hormonal alterations cannot be credited with all the symptoms, their persistence, and severity [7]. There is growing evidence in favour of a biopsychosocial model in which neuroendocrine changes are interacting with psychological and sociocultural factors to determine the menopausal experience [8]. Stress reactivity, coping capacity, emotional strength, and previous life events were found to have an effect on both the perception and distress of the symptoms during midlife [9]. Social-cultural discourses of ageing, femininity, productivity, and self-esteem can also be another contribution to emotional distress during menopause [10]. It is possible that over the life course, internalised ideals associated with the loss of attractiveness, personal value, or cognitive capacity may develop, and these may be more salient in this transition [11]. Such cognitive and emotional schemas have the potential to influence emotional regulation, stress reactivity, and attentional focus on bodily sensations so as to increase the burden of subjective symptoms [12].

Scholar and schema models of psychology suggest that strongly held beliefs acquired during early developmental and social life experiences still influence emotional and physiological reactions in adulthood [13]. In the menopause context, maladaptive belief constructions can be in interaction with physiological transformation to determine the interpretation of symptoms, emotional distress, and coping behaviour [14]. Such mechanisms align with recent studies in psychosomatic medicine that emphasise two-directional communication between emotional processing, autonomic regulation, and physical symptom expression [15]. Evidence-based interventions that are currently applied to manage menopausal symptoms comprise hormone replacement therapy, antidepressant medications, and structured psychological interventions like cognitive behavioural therapy and mindfulness-based interventions [16]. Though these treatments are useful for most women, they are largely focused on hormonal processes or conscious cognitive appraisal and may be inadequate to address deep-rooted belief processes or the processing of symptoms out of emotion [17]. As a result, a percentage of the women still complain of the persistent symptoms even after proper medical or psychological care [18].

Lesions in neuroscience and affective science have proved the ability of the adult brain to experience functional and structural modifications by means of neuroplastic mechanisms [19]. Experimental studies of memory reconsolidation and stress-system regulation provide a reason to believe that emotionally salient experiences may alter neural circuits and have a role in the perception of symptoms by disrupting the autonomic and affective reactions. These results have underpinned the growing study concern on integrative, faith-based therapies to alter maladaptive emotional and thinking patterns in an organised clinical paradigm.

Amongst the health conditions of women, biopsychosocial and neurocognitive-based integrative interventions are studied as adjunctive approaches, becoming more popular as a supportive treatment to the complex mind-body interactions of conditions. Nevertheless, there is a relative paucity of empirical studies evaluating structured psychological interventions targeting emotional regulation and cognitive appraisal in menopausal symptom management. Such methods should be carefully and closely screened clinically to ascertain their possible relevance to current conventional therapies. The present study evaluates a structured psychological intervention within a clinical research framework, emphasising measurable changes in menopausal symptom severity and short-term follow-up outcomes. By situating the intervention within established biopsychosocial and neurocognitive models, the study aims to contribute clinically relevant evidence to the evolving field of integrative menopause care.

Objective of the study

The primary objective of this study was to evaluate the association between a structured, non-pharmacological psychological intervention and within-subject changes in symptom severity across psychological, cognitive, and physical domains in perimenopausal and menopausal women, assessed from baseline to immediately post-intervention. Secondary objectives included descriptively examining symptom severity trends at one-week and four-week follow-up, assessing the feasibility and acceptability of delivering a single-session structured psychological intervention in a real-world setting, and exploring qualitatively reported changes in emotional regulation and cognitive clarity following the intervention.

## Materials and methods

Study design

This study was designed as a prospective, single-arm, non-randomised, uncontrolled exploratory clinical pilot study intended to assess feasibility, acceptability, and preliminary symptom outcomes. The study was conducted as a mixed-methods, exploratory clinical trial that aimed to investigate the impact of a structured psychological intervention on menopausal symptoms. The design combined quantitative symptom ratings with qualitative self-report ratings to address changes in the emotional, cognitive, and physical domains. A repeated-measures design was utilised, with measurements taken at baseline, immediately after the intervention, and in the short-term follow-up. The trial was non-randomised and uncontrolled, which was also planned as a pilot trial and aimed to determine its feasibility, acceptability, and initial clinical results in a real-world therapeutic setting in line with an integrative medicine study. The study was conducted under the supervision of BCCR Limited (Belief Coding Cognitive Rewiring), a behavioural wellness organisation operating in the United Kingdom.

Participant recruitment and eligibility

Participants were recruited between February 2025 and May 2025 in the United Kingdom via women's health clinics, online menopause information communities, and professional wellbeing networks. Intervention delivery and follow-up assessments were completed by September 2025. Respondents were recruited between February and May 2025 in the United Kingdom via women's health clinics, online communities for menopause information, and professional wellbeing networks. The eligibility criteria included women between 39 and 60 years old who reported either emotional, cognitive, or physical symptoms that they attribute to perimenopause or menopause and exhibited moderate or severe symptoms with a standardised symptom scale. The exclusion criteria were a diagnosed severe psychiatric condition, recent surgical menopause, pregnancy or postnatal status and the use of a psychiatric drug or psychotherapy. The method of purposive sampling was applied to make sure that different symptom profiles were represented within specific therapeutic parameters.

Ethical considerations and consent

Ethical considerations for human subjects were followed throughout all study procedures. Before enrolment, participants provided written informed consent after receiving a thorough explanation of the study objectives, methods used, potential risks, and their right to withdraw from the study at any time without penalty. The privacy and confidentiality of the data were guaranteed by the anonymisation and safe storage according to the relevant data protection laws. The participants were given a standardised intake checklist that included a record of appropriate medical history, menopause status, and current treatments. Ethical supervision emphasised the safety, psychological health and transparency of the study participants.

Intervention description

The intervention consisted of a single structured, non-pharmacological psychological session delivered individually (one-to-one) according to a predefined clinical protocol. Each session lasted approximately 60-75 minutes. The session was designed to assist emotional regulation and cognitive reframing using guided relaxation, focused concentration and guided reflective strategies. The predefined protocol incorporated guided relaxation techniques, focused attention exercises, and structured reflective strategies aimed at exploring emotional responses, cognitive appraisals, and stress-related reactions associated with participants’ predominant menopausal symptoms. Participants were guided to explore emotional responses, cognitive appraisals, and stress-related reactions associated with their predominant menopausal symptoms. Even though every single session focused on a specific target symptom, the protocol enabled the exploration of emotionally related experiences. The intervention was not pharmacological but focused on the continuity of the intervention, psychological containment, and the comfort of the participants.

Data collection procedures

A study-specific 19-item numerical rating scale was used to assess menopausal symptom severity across physical, emotional, cognitive, and sleep-related domains. Each item was rated on a 0-10 scale (0 = absent, 10 = maximum severity). As this instrument has not been formally psychometrically validated, future studies should incorporate recognised validated tools, such as the Greene Climacteric Scale or MENQOL questionnaire, alongside such ratings. The severity of the symptoms was assessed on a numerical scale at four time points: pre-intervention, immediately post-intervention, one week post-intervention, and four weeks post-intervention. The qualitative data were collected via structured post-session self-report data and follow-up interviews with participants via telephone or video conferencing. These qualitative indicators examined perceived changes in symptoms, emotional awareness, and the incorporation of coping mechanisms after the intervention.

Outcome measures and follow-up

The main finding was a time-varying change in symptom severity across the assessed domains. The secondary outcomes were emotional regulation, as reported by the participants, cognitive clarity, as reported by the participants, and how they perceived it to affect their daily functioning. A one- and four-week follow-up analysis was conducted to assess the short-term sustainability of the observed change and to record ongoing symptom patterns. The combined quantitative and qualitative outcome measures were chosen to include both quantitative and qualitative outcomes that are clinically measurable and subjective, respectively, in the integrative menopause care.

Ethical approval

Ethical approval for this study was obtained from the Ethics Review Committee of J.K. Multispeciality Hospital, Sanand, Ahmedabad, India (Approval No. 221025; issued 22 October 2025). The study was conducted under the operational oversight of BCCR Limited (Belief Coding Cognitive Rewiring), a behavioural wellness organisation registered in the United Kingdom, which served as the legal sponsor and data controller. Intervention sessions were delivered online via secure video conferencing, and no NHS sites, NHS staff, or NHS patients were involved. Accordingly, approval from a UK NHS Research Ethics Committee was not required under UK Health Research Authority guidance for research conducted outside NHS settings. Data were processed in compliance with the UK General Data Protection Regulation (UK GDPR) and the Data Protection Act 2018. All participants provided written informed consent before enrolment. The study was not prospectively registered in a clinical trial registry; this is acknowledged as a limitation of this exploratory pilot study, and future controlled trials should be prospectively registered.

Sample size calculation

A priori statistical power analysis was performed using G*Power software version 3.1.9.7 (Heinrich Heine University Düsseldorf, Düsseldorf, Germany) to estimate the minimum sample size required to detect within-subject changes using a paired-samples t-test. Assuming a medium effect size (Cohen’s d = 0.50), an alpha level of 0.05, and a statistical power of 0.80, the minimum required sample size was calculated to be 34 participants. The final analysed sample included 64 participants, exceeding the required threshold and providing adequate statistical power for detecting intervention-related changes.

Statistical analysis

Quantitative analyses were conducted using paired-samples t-tests to compare baseline and immediate post-intervention symptom severity scores. Effect sizes were calculated using Cohen’s d. To account for multiple comparisons across the 19 symptom domains, p-values were adjusted using the Benjamini-Hochberg false discovery rate (FDR) procedure. Descriptive statistics were calculated for all symptom domains across the four assessment time points. All statistical analyses were performed using Python version 3.13.12 (Python Software Foundation, Beaverton, OR, USA) with scientific computing libraries, including NumPy, Pandas, SciPy (stats module), StatsModels, and Pingouin. Data visualisation was generated using Matplotlib and Seaborn.

## Results

All 64 participants who completed the intervention and all the planned tests were included in the final analysis. The severity of the symptoms was measured at four time points: baseline (pre-intervention), immediately after the intervention, one week after the intervention, and four weeks after the intervention. Quantitative analysis revealed statistically significant within-subject reductions in symptom severity across several menopausal domains following the intervention. Psychological and cognitive domains, such as anxiety, mood instability, fatigue, and subjective cognitive impairment, recorded the highest severity scores at baseline. A moderate-to-high baseline severity was also shown by vasomotor symptoms, such as hot flushes and night sweats, and musculoskeletal pains. Following the intervention, symptom severity scores fell across all measured domains, with the largest reductions observed in psychological and cognitive symptoms. Descriptive trends at one-week and four-week follow-up assessments suggested no general return to baseline levels; however, these follow-up observations are reported descriptively and were not subjected to formal longitudinal inferential testing.

Statistical analysis and sample characteristics

Paired t-tests were used to conduct the statistical analyses to compare the scores of the symptom severity at different time points. Cohen’s d was used to measure the magnitude of change by determining the effect sizes. Multiple-comparison adjustments were made on the basis of the Benjamini-Hochberg FDR procedure. Statistical significance was considered at p < 0.05. The sample size of the study was 64 women aged between 39 and 60 years who reported having moderate-to-severe menopausal or perimenopausal symptoms at baseline. The average baseline symptom scores across the 19 domains assessed ranged from 2.5 to 7.2 on a 0-10 numerical scale, indicating moderate-to-severe symptom burden at baseline.

Primary outcome analysis

Pre- to post-intervention comparisons revealed statistically significant within-subject reductions in symptom severity across all 19 assessed domains. The largest effect size magnitude was observed for subjective cognitive impairment (brain fog; d = 1.28). Large effect size magnitudes were also observed for anxiety, mood swings, sleep disturbances, fatigue, depression, joint pain, and weight-related concerns. Vasomotor symptoms and urinary and skin-related symptoms showed moderate effect size magnitudes. Light-headedness and new facial hair demonstrated smaller but statistically significant pre-post differences. Note that several confidence intervals for moderate and smaller effect sizes crossed zero, indicating reduced precision for those outcomes and warranting cautious interpretation. Table 1 presents effect sizes, confidence intervals, and adjusted significance levels across all symptom domains.

**Table 1:** Pre- and post-intervention symptom scores (n = 64) The post represents measurements obtained immediately after the intervention session. Statistical comparisons between baseline and post-intervention scores were performed using paired t-tests (df = 63). P-values were adjusted for multiple comparisons using the Benjamini-Hochberg false discovery rate procedure. Clinical significance was classified based on Cohen’s d effect sizes as small (≥0.2), moderate (≥0.5), large (≥0.8), and very large (≥1.2). Symptom severity was assessed on a 0-10 numerical scale, where higher scores indicate greater severity; a reduction in mean score reflects improvement.

Symptom	Baseline Mean ± SD	Post Mean ± SD	t	df	p-value	Adjusted p	Cohen’s d	95% CI	Clinical Significance
Brain Fog	7.2 ± 1.9	4.6 ± 2.0	10.24	63	<0.001	<0.001	1.28	[0.35, 2.21]	Very Large
Anxiety	6.8 ± 1.8	4.3 ± 1.9	9.28	63	<0.001	<0.001	1.16	[0.51, 1.82]	Large
Mood Swings	6.6 ± 1.7	4.2 ± 1.9	8.80	63	<0.001	<0.001	1.10	[0.41, 1.80]	Large
Sleeplessness	6.9 ± 2.0	4.4 ± 2.1	8.64	63	<0.001	<0.001	1.08	[0.40, 1.76]	Large
Fatigue	6.7 ± 1.9	4.3 ± 2.0	8.48	63	<0.001	<0.001	1.06	[0.34, 1.77]	Large
Depression	6.4 ± 1.8	4.1 ± 1.9	8.32	63	<0.001	<0.001	1.04	[0.30, 1.78]	Large
Joint Pain	6.3 ± 2.0	4.1 ± 2.1	8.00	63	<0.001	<0.001	1.00	[0.23, 1.76]	Large
Weight Gain Concern	6.0 ± 1.8	4.0 ± 1.9	7.60	63	<0.001	<0.001	0.95	[0.21, 1.68]	Large
Backache	6.1 ± 1.9	4.3 ± 2.0	7.20	63	<0.001	<0.001	0.90	[0.25, 1.55]	Large
Irritability	6.2 ± 1.8	4.5 ± 1.9	7.04	63	<0.001	<0.001	0.88	[0.14, 1.62]	Large
Muscle Pain	5.9 ± 2.0	4.4 ± 2.0	6.48	63	<0.001	<0.001	0.81	[0.11, 1.52]	Large
Decreased Libido	5.8 ± 2.1	4.2 ± 2.1	6.72	63	<0.001	<0.001	0.84	[-0.17, 1.86]	Large
Dry Skin	5.3 ± 1.9	4.2 ± 2.0	5.92	63	<0.001	<0.001	0.74	[0.03, 1.45]	Moderate
Breast Tenderness	4.9 ± 1.8	4.0 ± 1.9	4.88	63	<0.001	0.012	0.61	[0.03, 1.18]	Moderate
Hot Flushes	5.5 ± 2.0	4.6 ± 2.1	4.64	63	<0.001	0.018	0.58	[-0.20, 1.37]	Moderate
Urinary Frequency	5.1 ± 1.9	4.3 ± 1.9	4.56	63	<0.001	0.021	0.57	[0.08, 1.06]	Moderate
Night Sweats	5.4 ± 2.1	4.6 ± 2.1	4.24	63	<0.001	0.027	0.53	[-0.15, 1.21]	Moderate
Light-headedness	4.8 ± 1.8	4.2 ± 1.9	3.60	63	<0.001	0.034	0.45	[-0.23, 1.14]	Small
New Facial Hair	3.9 ± 1.6	3.4 ± 1.7	3.12	63	<0.001	0.045	0.39	[-0.26, 1.03]	Small

Clinical significance

Effect size magnitudes were classified following Cohen's (1988) conventions: small (≥ 0.2), moderate (≥ 0.5), large (≥ 0.8), and very large (≥ 1.2). It should be noted that Cohen’s d thresholds reflect standardised effect magnitudes and do not constitute evidence of clinical meaningfulness; formal minimum clinically important difference (MCID) thresholds were not established for this instrument. The largest effect sizes were observed for psychological symptoms (anxiety, depression, irritability, and mood instability) and the cognitive domain. Physical symptoms were more variable, with pain-related symptoms showing larger effect sizes than other physical symptoms. Table 2 shows the distribution of menopausal symptoms by effect size category.

**Table 2:** Clinical significance and effect size distribution

Effect Size Category	Cohen’s d Range	Number of Symptoms	Percentage (%)
Very Large Effect	> 1.2	1	5%
Large Effect	0.8–1.2	11	58%
Moderate Effect	0.5–0.8	5	26%
Small Effect	0.2–0.5	2	11%
Minimal Effect	< 0.2	0	0%

Responder analysis

The symptoms were grouped as responders when they registered a huge or very huge effect size (Cohen’s d ≥ 0.8). By this definition, 12 of 19 symptoms (63%) were able to qualify as responders. This was the threshold of all the psychological and cognitive symptoms. Symptoms of musculoskeletal pain, such as joint pain, muscle pain, and backache, also met the criteria as responders, even though they showed slower improvement patterns over time.

Symptom domain analysis

**Figure 1:**
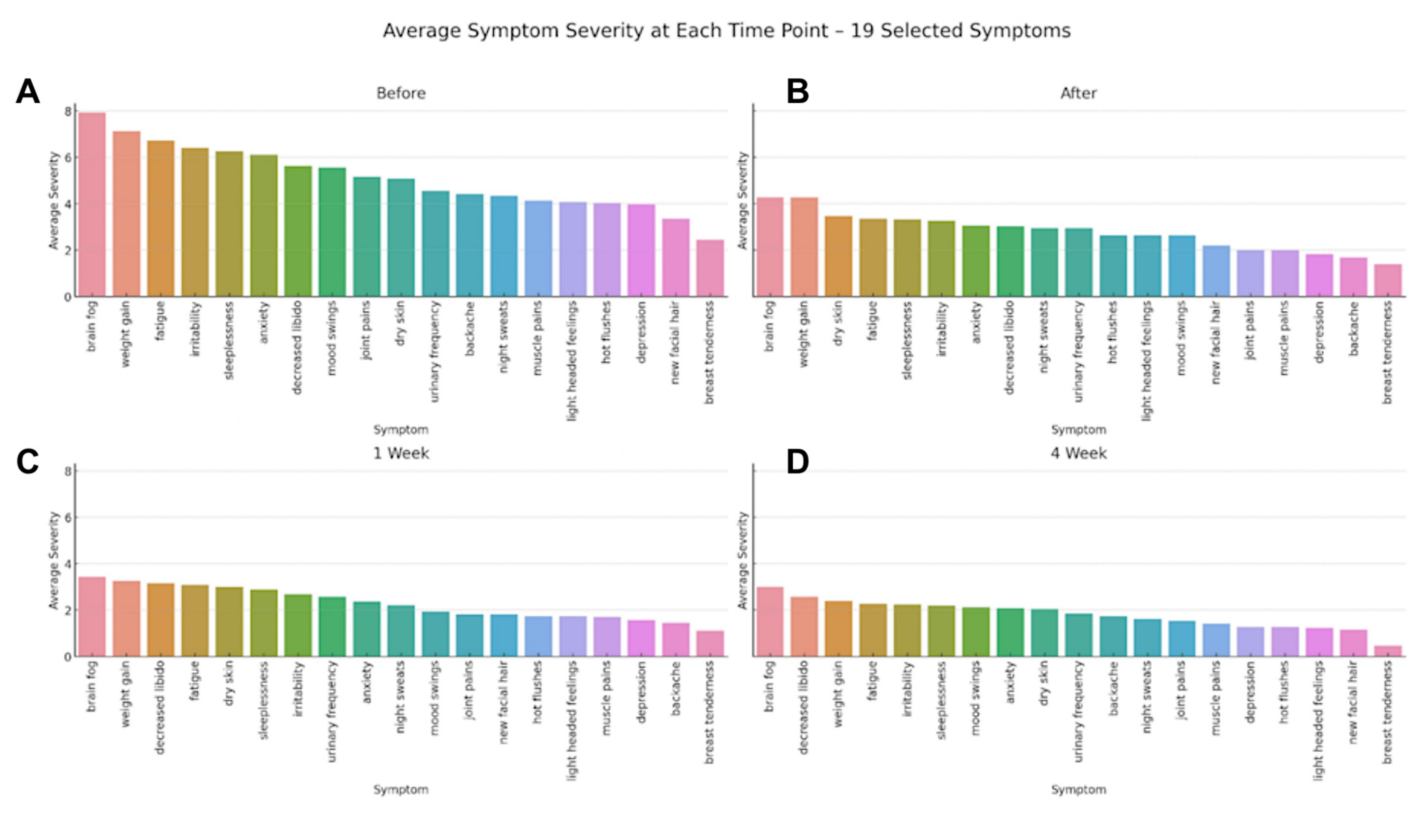
Average symptom severity across assessment time points (N = 64) Symptom severity was assessed using a 0–10 numerical rating scale, where 0 = no symptom and 10 = maximum symptom severity. Higher scores represent greater symptom burden. Values represent mean severity scores across four assessment time points: baseline (pre-intervention), immediately post-intervention, one-week follow-up, and four-week follow-up. (A) "Before" indicates baseline assessment conducted immediately prior to the intervention; (B) "After" indicates assessment conducted immediately following the session; (C) "1 week" indicates follow-up at seven days post-intervention; and (D) "4 week" indicates follow-up at 28 days post-intervention.

Analysis at the domain level showed similar response patterns. The effect size in the psychological symptom domain was d = 1.04, and this effect size was maintained across the follow-up measures. The cognitive domain had the highest mean effect size, which was mainly contributed to by an increase in fatigue and cognitive clarity. The mean effect size of the physical pain domain was d = 0.90. Vasomotor symptoms showed moderate improvement (mean d = 0.56), and sleep-related symptoms showed large and significant improvement over the follow-up period (mean d = 1.07). The results are represented in Figure 1 in the form of average severity scores of 19 menopausal symptoms at the baseline, right after intervention, one week, and four weeks after intervention.

Temporal response patterns

**Figure 2:**
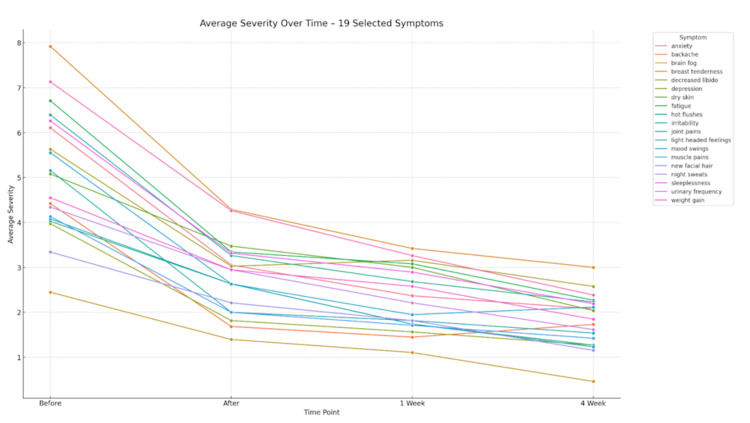
Average severity of menopausal symptoms over time (N = 64) Mean symptom severity scores were measured using a 0–10 numerical rating scale, where 0 = no symptom and 10 = maximum symptom severity. Higher scores indicate greater symptom burden. Data points represent the average severity scores for 19 menopausal symptoms assessed at baseline, immediately after intervention, at one-week follow-up, and at four-week follow-up. "Before" indicates baseline assessment conducted immediately prior to the intervention; "After" indicates assessment conducted immediately following the session; "1 week" indicates follow-up at seven days post-intervention; and "4 week" indicates follow-up at 28 days post-intervention.

Three response patterns in terms of time were noted. The pattern of psychological and energy-related symptoms was an immediate response, which improved rapidly, followed by stabilisation. The symptoms like cognitive impairment, sleeplessness and weight-related concerns showed a progressive-improvement trend, and they kept on improving in the follow-up. A stable-moderate response was noted in vasomotor and dermatological symptoms, but the improvement was moderate and sustained at regular time intervals. Figure 2 shows the mean severity scores for 19 menopausal symptoms at baseline, immediately post-intervention, at one-week and four-week follow-up.

Qualitative outcomes

Qualitative data were obtained from structured post-session self-reports and follow-up check-ins conducted at one week and four weeks following the intervention. Thematic analysis of participant responses revealed consistent patterns of perceived improvement across emotional and cognitive domains. Participants frequently reported increased emotional awareness, improved stress regulation, enhanced perceived cognitive clarity, and a greater sense of control over their menopausal symptom experience. Several participants described that psychological improvements, such as reduced anxiety or mood instability, appeared to precede reductions in perceived physical symptom distress. Reports of improved daily functioning, interpersonal interactions, and occupational performance were also noted during follow-up assessments. These qualitative findings complement the quantitative reductions in symptom severity observed across emotional and cognitive domains.

Statistical power and data quality

The sample size was also large enough (>80%) to have sufficient statistical power to identify medium-to-large effect sizes. Most outcomes fell within narrow confidence intervals, indicating precise estimates. None of the data anomalies was found, and complete data were available for all outcome measures across time points.

## Discussion

This exploratory clinical study assessed symptom changes following a structured, non-pharmacological psychological intervention on menopausal symptoms across psychological, cognitive, physical, sleep-related, and vasomotor domains. Statistically significant within-subject reductions in symptom severity were observed across all assessed domains, with the largest effect size magnitudes noted in psychological and cognitive domains. Descriptive follow-up data at one and four weeks suggested no general return to baseline, though formal longitudinal inference was not conducted, and these trends should be interpreted with caution. The pattern of large effect sizes in psychological and cognitive symptoms is consistent with well-known biopsychosocial models of menopause that focus on the interplay of neuroendocrine alteration and psychological mechanisms of symptom expression [20]. Anxiety, mood instability, depressive symptoms, fatigue, and subjective cognitive impairment all exhibited large to very large effect sizes and contribute to the relevance of interventions that focus on the emotional regulation and cognitive appraisal processes [15].

Physical symptoms showed a greater cross-interdomain response pattern. The effects were large on musculoskeletal pain symptoms, including joint pain, muscle pain and backache, and moderate but stable over time on the vasomotor symptoms of hot flushes and night sweats [21]. This heterogeneity has indicated the multifactorial aetiology of the menopausal symptoms and indicates differences in responses to non-pharmacological interventions [22]. This interpretation is also supported by the fact that specific patterns of responses are identified over time. Symptoms that were associated with psychology and energy showed quick improvement and stabilisation, but symptoms that were associated with the mind and sleep persisted to improve with the follow-up. Moderate improvements in vasomotor and dermatological symptoms were also observed, and no longer increased over time [18]. These data indicate that symptom patterns can be different depending on physiological and psychosocial processes behind these symptoms [23].

The responder analysis showed that a large or very large proportion of the symptoms were in the criteria of large or very large effect size, and all the psychological and cognitive symptoms were responders. These findings suggest that structured psychological interventions may warrant further evaluation as potentially feasible adjunctive approaches as an adjunctive tool in managing menopausal symptoms, especially in those women who have pronounced emotional or cognitive suffering [24]. Clinically, the findings are significant to the emerging literature on integrative and psychosomatic approaches in the health of women. Although pharmacological therapy remains the primary management approach for menopause, a proportion of women continue to experience persistent symptoms or are unable or unwilling to use hormonal treatments, highlighting the potential value of complementary strategies that address psychological and cognitive dimensions of symptom burden [25].

Menopausal symptoms frequently persist despite standard medical management, and some individuals are unable or unwilling to use pharmacological therapies. The findings of this exploratory study suggest that structured, non-pharmacological psychological interventions may offer a feasible adjunctive option for addressing psychological, cognitive, and selected physical symptoms in perimenopausal and menopausal individuals. While not intended to replace established treatments, such interventions may be considered within integrative or multidisciplinary care settings, particularly for patients experiencing prominent emotional or cognitive symptom burden.

Several important limitations must be considered when interpreting these findings. The single-arm, uncontrolled design does not permit causal attribution of observed changes to the intervention. In particular, the design is susceptible to regression to the mean (given enrolment on the basis of moderate-to-severe baseline symptoms), expectancy and placebo effects, demand characteristics (participants responding in line with perceived study aims), social desirability bias, and Hawthorne effects associated with the high-contact one-to-one format and immediate reassessment. These are especially relevant to the large effect sizes observed in self-reported psychological and cognitive domains. The study-specific outcome instrument has not been formally psychometrically validated, and all ratings were self-reported, introducing possible response bias. The four-week follow-up is insufficient to evaluate long-term durability. Future studies should employ randomised controlled designs with active or waitlist control conditions and longer follow-up periods. Incorporating recognised validated instruments (e.g., Greene Climacteric Scale, MENQOL) and objective physiological markers would substantially strengthen methodological rigour.

Limitations and future recommendations

This study can be viewed through some methodological issues. The non-randomised, exploratory design restricts the ability to draw inferences about causality and thus was not aimed at determining the comparative effectiveness but at determining feasibility and preliminary results. The assessment of the symptoms was based on self-report measures, the subjective experience, and was suitable to assess patient-centred outcomes in the menopause study. There was a short follow-up period (four weeks), which did not allow for concluding on the long-term sustainability of effects. Although the sample was large enough to identify medium-to-large effects, the generalisability of the results to all menopausal groups should be interpreted with caution.

Future research should build on these exploratory findings using randomised controlled designs with active or waitlist control conditions, enabling causal inference and direct comparison with established treatment modalities. Longer follow-up periods (minimum six months) are needed to assess symptom trajectory beyond the short-term window observed here. Outcome assessment should incorporate recognised validated instruments, such as the Greene Climacteric Scale or MENQOL, alongside objective physiological measures, to improve rigour and inter-study comparability. Investigation of multi-session interventions, symptom-specific protocols, and diverse participant populations would further inform how structured psychological approaches can be most effectively integrated into comprehensive menopause care.

## Conclusions

This exploratory study provides preliminary evidence suggesting that a structured psychological intervention is associated with significant reductions in menopausal symptom severity across psychological, cognitive, physical, sleep-related, and vasomotor domains. The findings support a biopsychosocial perspective on menopause management, emphasising that emotional regulation and cognitive processes may meaningfully influence symptom perception and overall distress during the menopausal transition. Reductions in self-reported symptom severity were observed following a single, non-pharmacological session, suggesting feasibility and potential short-term benefit within an integrative care framework; causal conclusions cannot be drawn from this uncontrolled design. The differential response patterns across symptom domains highlight the complex and multifactorial nature of menopausal symptoms, indicating that individualised approaches may be necessary to optimise outcomes. While psychological and cognitive symptoms demonstrated the most pronounced improvements, physical and vasomotor symptoms also showed measurable benefit, supporting the potential complementary role of structured psychological intervention alongside standard medical treatments. Given the exploratory and uncontrolled design of this study, further study is required to confirm these findings. Future investigations should employ randomised controlled methodologies, include longer follow-up periods, and examine multi-session or symptom-specific protocols to assess durability, generalisability, and clinical applicability in broader menopausal populations.

## Appendices

Menopause calculator

Client name: 

Session date:  

The severity of the symptom is scored as 0 = none, 10 = severe.

**Table 3:** Menopause questionnaire symptom scores across timepoints (n = 64)

	Score before BC	Score after BC	1 week check in	4 week check in
(date of check in)				
Overall feelings towards menopause				
Night sweats				
Hot flushes				
Irritability				
Depression				
Anxiety				
Mood Swings				
Sleeplessness				
Fatigue				
Backache				
Joint Pains				
Muscle Pains				
New Facial Hair				
Decreased libido				
Brain Fog				
Weight Gain				
Urinary frequency				
Breast tenderness				
Dry Skin				
Light headed feelings				

Client's signature:

Date:
